# Effects of Water-Soluble C_60_ Fullerenes on Rat Musculus Soleus Contraction Following Neurogenic Atrophy

**DOI:** 10.3390/molecules31132334

**Published:** 2026-07-03

**Authors:** Yuriy Prylutskyy, Dmytro Nozdrenko, Maksym Anhelov, Svitlana Prylutska, Olexandr Bezuh, Igor Vareniuk, Oleksii Sulyma, Vasyl Melenko, Kateryna Bogutska, Vsevolod Cherepanov, Mykola Petrovsky, Uwe Ritter, Jacek Piosik

**Affiliations:** 1 ESC “Institute of Biology and Medicine”, Taras Shevchenko National University of Kyiv, 01601 Kyiv, Ukraine; prylut@ukr.net (Y.P.); ddd@univ.kiev.ua (D.N.); maksym.anhelov@knu.ua (M.A.); psvit_1977@ukr.net (S.P.); tmo.hospital_mvs@ukr.net (O.B.); vareniuk_igor@yahoo.com (I.V.); bogutska_ki@knu.ua (K.B.); mykola.petrovsky@ukr.net (M.P.); 2 Kyiv Agrarian University, NAAS of Ukraine, 01010 Kyiv, Ukraine; 3 SI “The Institute of Traumatology and Orthopedics”, NAMS of Ukraine, 01601 Kyiv, Ukraine; sulymaoleksii@gmail.com (O.S.); melenkovasil@gmail.com (V.M.); 4 Institute of Physics, NAS of Ukraine, 03028 Kyiv, Ukraine; vvch2000@ukr.net; 5 Institute of Chemistry and Biotechnology, Technical University of Ilmenau, 98693 Ilmenau, Germany; uwe.ritter@tu-ilmenau.de; 6 Intercollegiate Faculty of Biotechnology, University of Gdansk, 80-309 Gdańsk, Poland

**Keywords:** water-soluble C_60_ fullerene, *musculus soleus*, neurogenic atrophy, biomechanical parameters of muscle contraction, blood biochemical indicators, histological analysis

## Abstract

Neurogenic atrophy is the most severe type of muscle atrophy. It can be caused by injury or disease of the nerve that connects to the muscle. Damage to the sciatic nerve (*nervus ischiadicus*) initiates molecular processes that lead to the transformation of muscle dysfunction into an atrophic state. Oxidative stress is one of the key factors that initiates skeletal muscle atrophy. Therefore, this study evaluates the effects of oral administration of water-soluble C_60_ fullerenes (daily dose: 1 mg/kg), as powerful antioxidants, on the contraction dynamics of the rat *musculus soleus* on days 15, 30, and 45 following neurogenic atrophy induced by transection of the *nervus ischiadicus.* Using biophysical (tensometric), biochemical, and histological analyses, we evaluated the biomechanical parameters of *musculus soleus* contraction (time of onset of muscle force response, integrated muscle power, maximum and minimum contraction forces), blood biochemical markers (concentrations of C-reactive protein, lactate, creatinine, and reduced glutathione, as well as superoxide dismutase and catalase activities), as well as histological and morphometric indicators of muscle damage in rats on days 15, 30, and 45 after injury induction. It was found that the use of water-soluble C_60_ fullerenes improves the contractile activity of the *musculus soleus* after neurogenic atrophy and has a time-dependent nature. Specifically, by day 45 of the experiment, the maximum therapeutic effect reached 23–35 ± 2% for the biomechanical parameters of muscle contraction, and the biochemical blood parameters have nearly approached the control values. Finally, histological analysis confirmed a significant reduction in signs of destruction in muscle fibers and the level of fibrosis in the *musculus soleus*. These findings suggest the potential application of water-soluble C_60_ fullerenes in the treatment of pathological conditions of the muscular system arising from peripheral nerve injury.

## 1. Introduction

Sciatic nerve injury is among the most common lesions of the peripheral nervous system. It leads to motor and sensory deficits and the development of chronic pain, significantly diminishing patients’ quality of life [1]. Although the sciatic nerve possesses an intrinsic capacity for endogenous regeneration, the recovery of motor and sensory functions remains limited, highlighting the need for new and effective therapeutic strategies to address this major clinical challenge [2].

An inevitable consequence of sciatic nerve injury is the development of neurogenic muscle atrophy, leading to reduced interaction between motoneurons and skeletal muscle fibers [3]. Sciatic nerve injury results in a 90% inhibition of skeletal muscle mass [4]. The rate and extent of muscle mass loss depend on both the type of muscle (slow or fast) and the degree of their inactivity. There is also a significant reduction in the diameter of muscle fibers, which is especially pronounced in slow type I fibers, decreased sarcomere density, proliferation of connective and adipose tissue (interstitial fibrosis) and degenerative ultrastructural changes in the mitochondria and sarcoplasmic reticulum. These alterations lead to a decrease in the cell’s energy potential, and also functional capacity and a loss of neurotrophic control over muscle tissue [5]. Additionally, in rats, following sciatic nerve transection, approximately one-third of spinal ganglion neurons and up to half of spinal motor neurons die [4]. Without proper trophic innervation, skeletal muscles also undergo remodeling [6]: myofibrils decrease in size, being compressed by surrounding myofibrils, while others relocate myonuclei to the center of myofibrils. The observed deficit in muscle strength may be caused by a reduction in the number of regenerating axons [7,8]. In addition to skeletal muscles, surrounding tissues undergo remodeling. Specifically, there is an accumulation of fibroadipose tissue in the absence of a significant increase in the number of macrophages and muscle stem cells [9]. However, it is important to recognize that macrophages play a dual role in the development of fibrosis: they can both promote the accumulation of scar tissue and contribute to its resolution. Pro-inflammatory macrophages secrete cytokines such as TNF-α, IL-1, and IL-12, as well as reactive oxygen species, which activate collagen formation processes. Profibrotic macrophages produce TGF-β, transforming fibroblasts into myofibroblasts that synthesize collagen and extracellular matrix, thereby forming scar tissue. If this process is not properly regulated, functional tissue is eventually replaced by connective tissue. Finally, fibrolytic macrophages synthesize matrix metalloproteinases that degrade excess collagen and stimulate the apoptosis of surplus myofibroblasts by engulfing their remnants. Fibrosis results from a disruption of this delicate balance [10].

Thus, skeletal muscle fibers remain denervated for an extended period, which leads to the activation of pathways associated with atrophy and, ultimately, loss of muscle function. Prolonged muscle denervation also causes motor nerve endplates to become non-permissive to axonal innervation, resulting in a non-functioning synapse [11]. Consequently, by the time nerve endings reach the muscle for reinnervation, it has become severely atrophied and unable to “accept” it.

Modern therapeutic strategies aimed at maintaining muscle mass during reduced neural activity involve the use of physical exercise and pharmacological agents capable of slowing the progression of the pathological process. Non-pharmacological strategies based on electrical stimulation that mimics nerve impulses help maintain muscle fiber volume and blood microcirculation [12]. Pharmacological strategies involve several promising groups of drugs [13]: selective androgen receptor modulators and steroids help retain nitrogen in tissues and stimulate protein synthesis; myostatin inhibitors accelerate recovery processes; β2-adrenergic receptor agonists promote skeletal muscle hypertrophy even under conditions of limited mobility; leucine stimulates the mTOR signaling pathway, which is directly responsible for muscle protein synthesis; creatine therapy enhances the energetic potential of cells and promotes water retention in muscle fibers, thereby slowing their degradation. However, these approaches do not provide a fully long-term or sustainable effect [14]. Currently, there is also no reliable surgical method for restoring damaged nerve trunks (tactile discriminative ability) [14].

Post-traumatic nerve regeneration is a complex biological process, the positive outcome of which depends on many factors, such as the survival of nerve cells, the rate of axon regeneration, the type of injury, the type of nerve and the extent of its damage, the patient’s age, and adherence to a physical training regimen. A significant challenge is cortical functional reorganization, which occurs due to misdirected axon growth during post-traumatic regeneration [15].

Note that inflammation is an inevitable process during sciatic nerve injury and recovery. “Suppression” of the inflammatory process reduces the degree of nerve damage and the level of neuropathic pain, promoting its rapid restoration [16]. Current pharmacological agents can modulate signaling pathways associated with inflammation. Specifically, they inhibit the activation of NF-κB signaling pathways and regulate the expression of TNF-α and IL-1β cytokines, thereby reducing nerve inflammation and local tissue damage [17]. However, their therapeutic effects are associated with the development of several side effects on the body [18].

Oxidative stress plays a significant role in post-traumatic nerve regeneration and the development of inflammatory processes [19]. Free radicals can induce neuronal apoptosis through the mitochondrial pathway and regulate the expression of apoptotic genes involved in the sensitization of neuropathic pain [20]. Inflammatory processes arising from peripheral nerve damage led to the accumulation of free radicals. The use of antioxidants in this context promotes neuroregeneration and indirectly achieves an analgesic effect by modulating pain signal transmission pathways [21]. Specifically, it has been shown that the application of a B-vitamin complex reduces the expression of pro-inflammatory cytokines in peripheral nerve injury, which facilitates the regression of inflammation [22]. The primary mechanism by which vitamin E restores sciatic nerve function after crush injuries involves the regulation of oxidative stress pathways [23]: vitamin E, by integrating into the cell membrane, neutralizes free radicals and interrupts the chain reaction of lipid peroxidation. This prevents the death of nerve regions that were not damaged during the mechanical injury. In addition, vitamin E slows Wallerian degeneration by reducing the activity of inflammatory enzymes and cytokines, thereby limiting the affected area and preventing degeneration from affecting the neuronal cell body. As a result, vitamin E promotes the formation of a loose matrix rather than dense scar tissue at the site of injury [24].

Furthermore, the combination of the antioxidant pregabalin and B vitamins significantly reduced the inflammatory response and Wallerian degeneration of the sciatic nerve [25]. It has been shown that carnosine, a pleiotropic dipeptide with antioxidant properties, accelerates the recovery of injured sciatic nerves [26]. In this case, the severity of Wallerian degeneration, myelin fiber density, myelin sheath thickness and diameter, as well as ultrastructural changes in myelinated axons, were reduced. Finally, biochemical parameters of nerve tissue (malondialdehyde and superoxide dismutase (SOD)) altered by compression injury were restored, and muscle mass approached normal levels.

It has been established that C_60_ fullerenes, as representatives of the third allotropic form of carbon, effectively capture and inactivate free radicals in both in vitro and in vivo systems [27,28,29]. Notably, a single C_60_ molecule can simultaneously bind 34 methyl radicals [30]. Data [31,32] indicate complex mechanisms of interaction between nanostructures and biological systems, including the regulation of redox balance, cellular signaling pathways and local microenvironment of damaged tissues, and extracellular matrix remodeling. Distant organ damage in the lung and heart tissues caused by lower-extremity ischemia–reperfusion injury can be significantly reduced by C_60_ fullerenol (C_60_(OH)_n_) [33]. Long-term administration of C_60_ fullerenol improved behavioral and molecular markers of Alzheimer’s disease in mice [34]. C_60_ fullerenol also has protective effects against skeletal muscle damage resulting from ischemia–reperfusion in diabetic rats [35]. Oral administration of C_60_ fullerene reduced the number of inflammatory cells, edema formation, and hydropic degeneration in damaged rat muscle tissue [36]. In several of our previous studies on animal models [37,38,39], oral administration of C_60_ fullerene aqueous solution (C_60_FAS) at a low effective dose (1 mg/kg) following the induction of skeletal muscle damage of various origins produces significant therapeutic effects. For instance, treatment with C_60_FAS nearly normalized the biomechanical parameters of *musculus soleus* contraction and the biochemical blood parameters in rats with chronic glyphosate intoxication [37]. The recovery time of the rats’ *musculus soleus* after atrophy induced by Achilles tenotomy was reduced by more than 30% following C_60_FAS administration [38]. Additionally, C_60_FAS decreased the stiffness of the injured rat *musculus gastrocnemius* by slowing the development of fibrosis and suppressing inflammatory processes within the muscle, thereby contributing to a shorter post-traumatic recovery period [39]. More recently, it was found that C_60_FAS restored the contractile activity of the rat *musculus gastrocnemius* (fast-twitch muscle) by day 30 after neurogenic atrophy caused by damage to the sciatic nerve (*nervus ischiadicus*) [40]. These findings prompted us to expand our research to analyze in detail the impact of C_60_FAS on the contraction of the rat *musculus soleus* (a slow-twitch muscle) at 15, 30, and 45 days after neurogenic atrophy caused by damage to the *nervus ischiadicus*, using biophysical (tensometric), biochemical methods, and histological methods to analyze blood and muscle tissue, respectively.

## 2. Results and Discussion

### 2.1. Atomic Force Microscopy (AFM) Characterization of C_60_FAS

**Figure 1:**
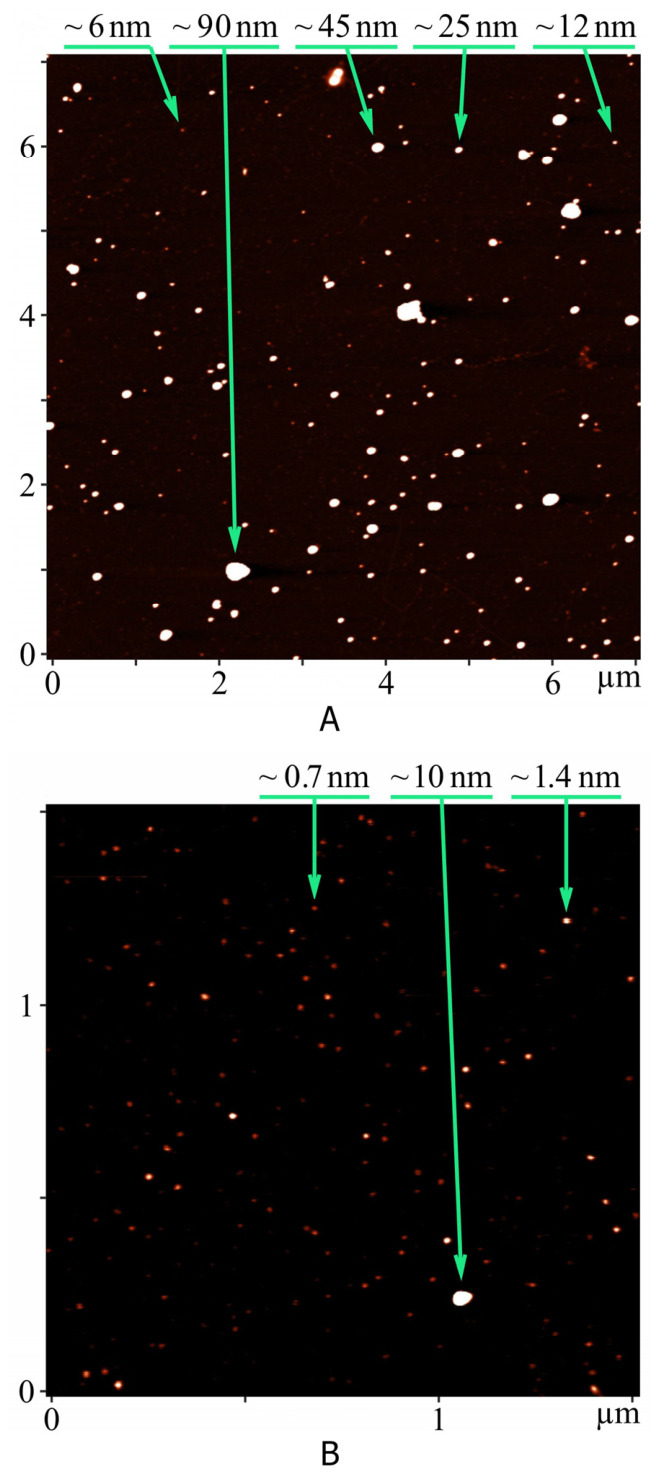
AFM images of C_60_ fullerene layer deposited from C_60_FAS onto a mica substrate. The distance values near the arrows indicate the heights of the nanoparticles. (**A**,**B**) show 2 different shots of the same nanoparticles.

As shown in Figure 1A, the structure of the C_60_ fullerene layer consists of objects with heights ranging from a few nanometers up to 100 nm. These objects are aggregates of C_60_ molecules, characteristic of C_60_FAS [41]. The size distribution of the aggregates is polydisperse, with a broad peak between 12 and 40 nm.

To detect smaller aggregates, AFM images were recorded with the inter-dot/inter-line spacing reduced to 1.5 nm, and measurements were taken in areas free of large aggregates. This image (Figure 1B) shows point objects with a height of approximately 0.7 nm, corresponding to individual C_60_ molecules, as well as aggregates with a height of approximately 1.4 nm. Thus, according to the AFM data, the characterized solution contained C_60_ fullerene in both molecular low-aggregated and highly aggregated forms.

### 2.2. Biomechanics of Musculus Soleus Contraction

High-frequency stimulation of peripheral afferent nerves that form monosynaptic contacts with motoneurons promotes effective summation of successive action potentials and sustained depolarization of the cell membrane. When pathological processes develop, impairing nerve function, a characteristic adaptive decrease in the conduction time of the stimulus through the nerve tissue occurs, accompanied by an increase in the number of tetanic contractions with minimal relaxation periods between them. Changes in this parameter serve as an important marker of pathological processes in neuromuscular preparations when stimulation signals approaching physiological values are applied [38].

**Figure 2:**
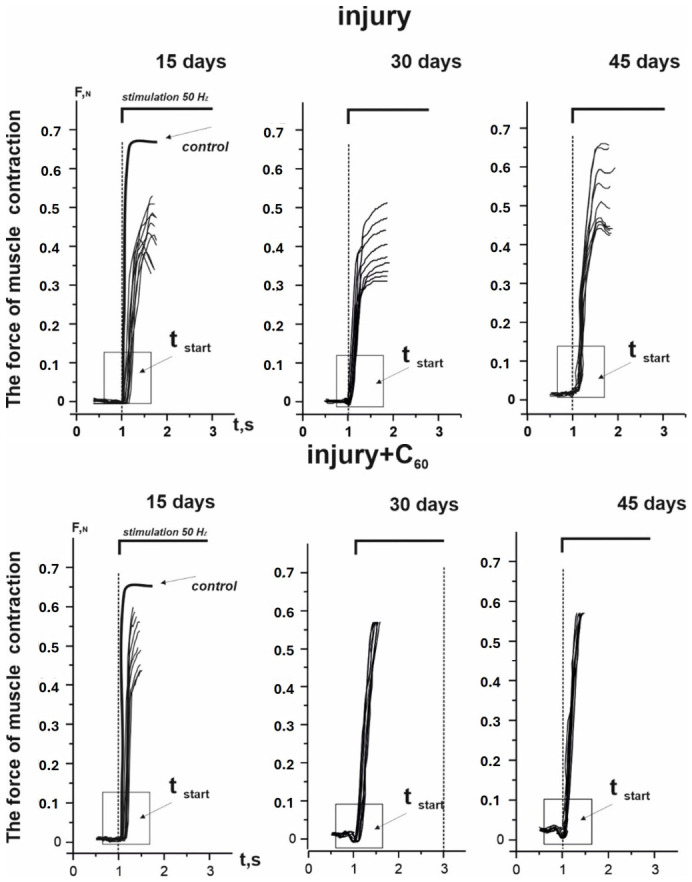
The dependence of rat *musculus soleus* contraction force (F, N) on time (t, s), induced by 10 consecutive non-relaxation electrical stimulation pulses at a frequency of 50 Hz: control—mechanograms of the control group animals; injury and injury + C_60_—mechanograms obtained on days 15, 30, and 45 after *nervus ischiadicus* injury and with daily oral administration of C_60_FAS (1 mg/kg), respectively; t_start_—time of onset of the *musculus soleus* force response.

**Figure 3:**
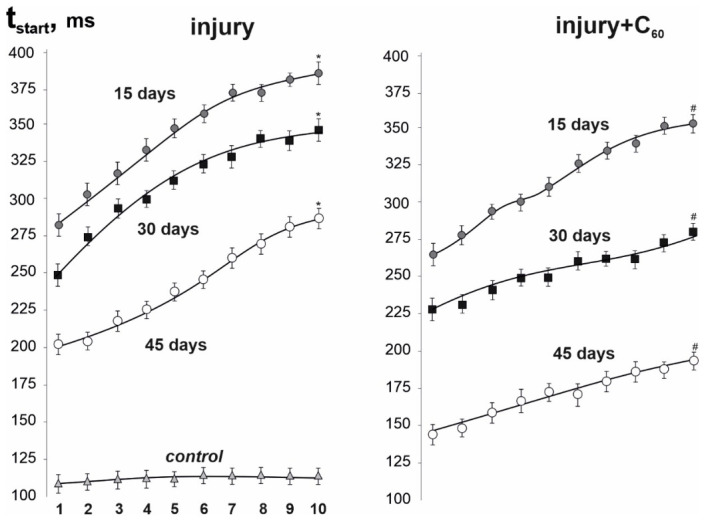
Time of onset of the *musculus soleus* force response in rats (t_start_, ms), induced by 10 (1, 2…10) consecutive non-relaxation electrical stimulation pulses at a frequency of 50 Hz: control—control group of animals (*p* < 0.05); injury and injury + C_60_—kinetic curves obtained on days 15, 30, and 45 after *nervus ischiadicus* injury and with daily oral administration of C_60_FAS (1 mg/kg), respectively; * *p* < 0.05 relative to the control group; ^#^ *p* < 0.05 relative to the injury group.

After nerve injury and partial regeneration, a reduction in the conduction time of stimulation impulses was observed (Figure 1). Analysis of the mechanograms of *musculus soleus* contraction revealed a significant delay in the onset of the force response on day 15 after injury (Figure 2 and Figure 3). The delay increased with each successive muscle contraction, reaching 278 ± 7 ms and 387 ± 10 ms on the first and tenth contractions, respectively (compared to 105 ± 5 ms and 110 ± 5 ms in controls) (Figure 3). On post-injury days 30 and 45, this parameter reduced to 248 ± 5 ms and 342 ± 4 ms, and 205 ± 3 ms and 288 ± 5 ms, respectively, for the first and tenth contractions. However, these values remained more than twice those of the controls (Figure 3). Based on these data, it can be concluded that neuropathic and myopathic changes persist, associated with the inability to process successive stimulation impulses without significant physiological impairments, even 45 days after nerve injury.

Administration of C_60_FAS for 15 days post-nerve injury reduced the onset time of the *musculus soleus* force response to 262 ± 5 ms and 351 ± 7 ms for the first and tenth contractions, respectively, which was 6–9 ± 1% lower than in the injury group. On days 30 and 45 of the experiment, these values were 228 ± 3 ms and 276 ± 4 ms, and 144 ± 3 ms and 183 ± 4 ms, respectively. Thus, the positive effect of C_60_FAS resulted in reductions of 8–19 ± 1% and 30–36 ± 2% on days 30 and 45, respectively, compared to the injury group. These results suggest that water-soluble C_60_ fullerenes may suppress fatigue processes in actively atrophied muscle, consistent with previous observations [42].

**Figure 4:**
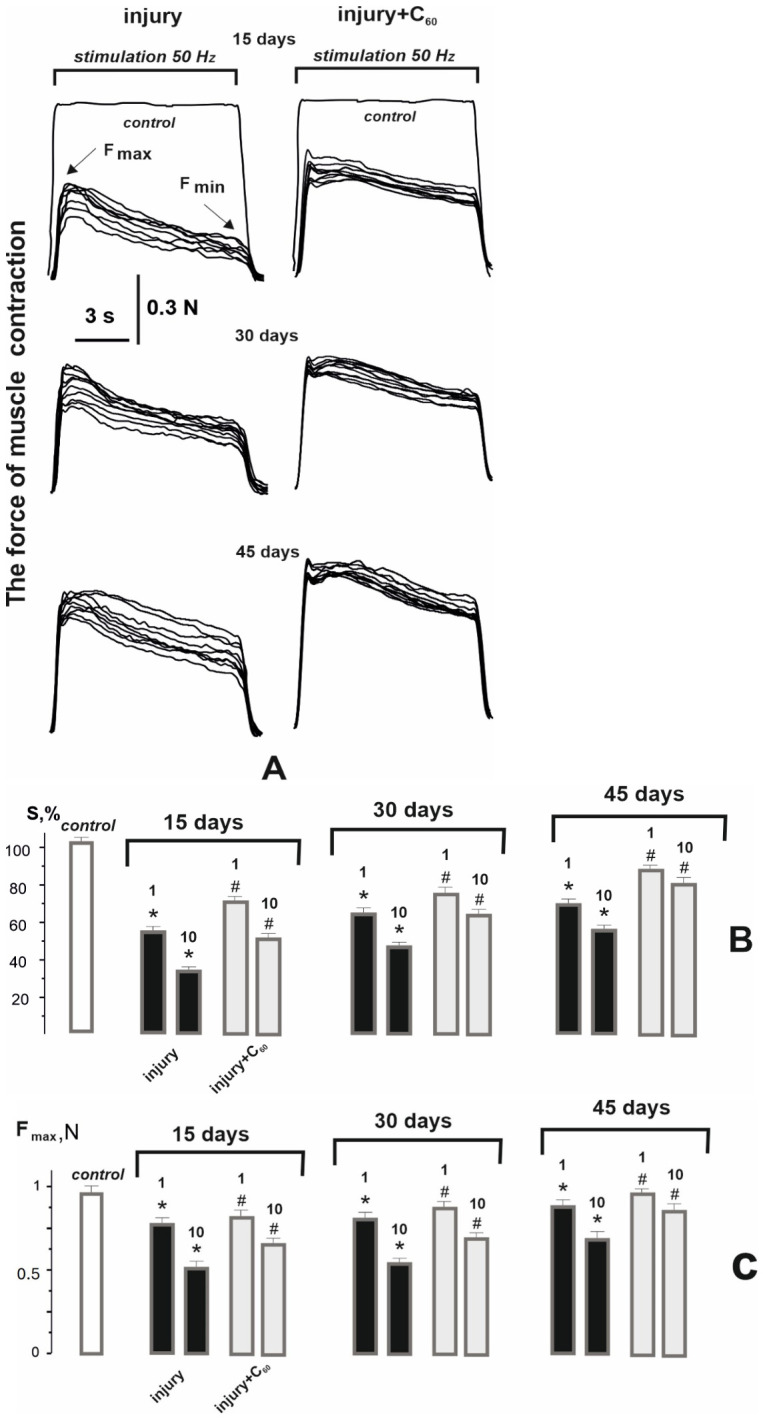

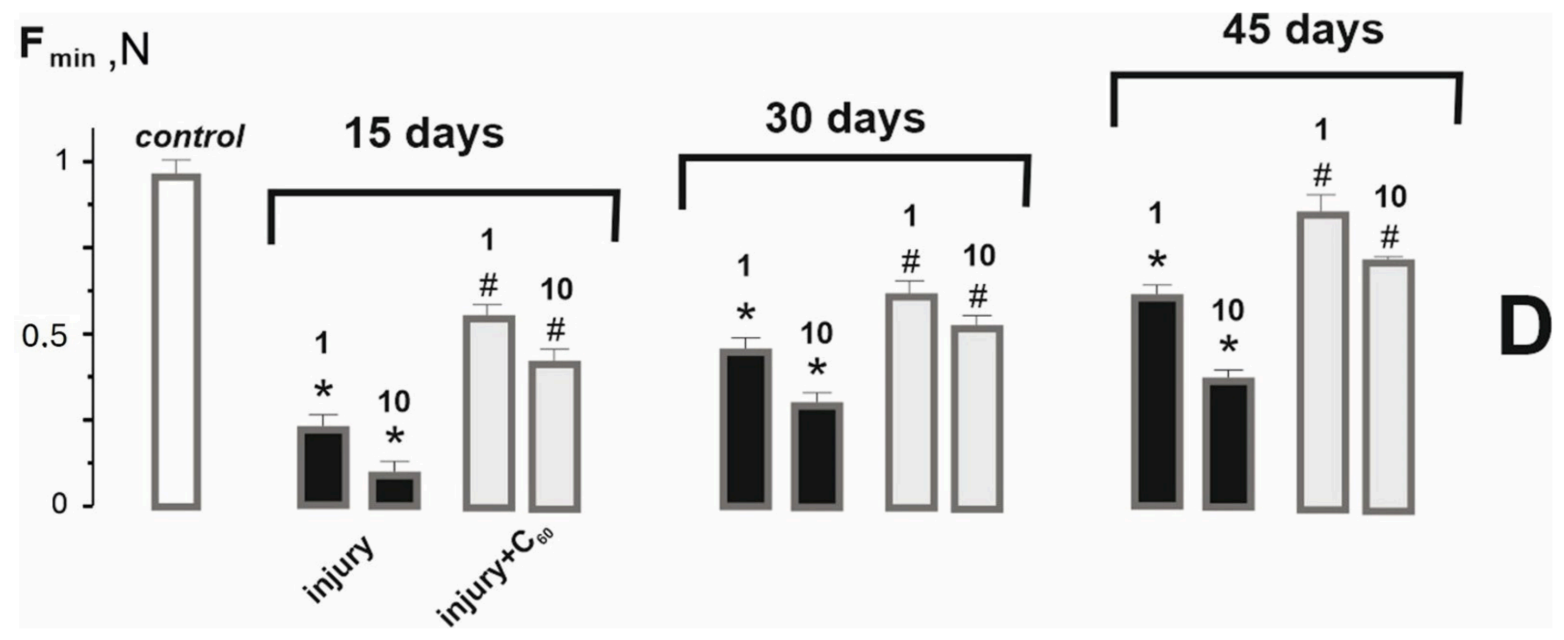
Contractile activity of the *musculus soleus* in rats induced by 10 consecutive non-relaxation trains of electrical stimulation at 50 Hz: (**A**) original mechanograms (the force of *musculus soleus* contraction); (**B**) integrated muscle power (S, calculated relative to the control, taken as 100%); (**C**) maximum muscle contraction force (F_max_, N); (**D**) minimum muscle contraction force (F_min_, N). Control (white): control group of animals (*p* < 0.05); injury (black) and injury + C_60_ (gray): results obtained on days 15, 30, and 45 after *nervus ischiadicus* injury and with daily oral administration of C_60_FAS (1 mg/kg), respectively; 1 and 10: first and tenth *musculus soleus* contractions, respectively. * *p* < 0.05 relative to the control group; ^#^ *p* < 0.05 relative to the injury group.

Changes in the levels of integrated muscle power, as well as maximum and minimum contraction force, are important markers of pathological processes in myocytes, including those caused by neurogenic atrophy (Figure 4).

The integrated muscle power value enables assessment of its functional activity within the equilibrium system of “contraction force–external load,” serving as a physiological analogue of the overall working capacity of the muscular system [37,38,39,40,42].

On day 15 after nerve injury, integrated muscle power significantly reduced by 55 ± 3% and 32 ± 2% for the first and tenth contractions, respectively, compared to the control group (set at 100%) (Figure 4B). On days 30 and 45, these values were 63 ± 4% and 48 ± 3%, and 70 ± 4% and 51 ± 3%, respectively, for the first and tenth muscle contractions (Figure 4B).

Administration of C_60_FAS increased integrated muscle power to 70 ± 5% and 50 ± 3%, 75 ± 4% and 62 ± 4%, and 88 ± 6% and 80 ± 5% on days 15, 30, and 45, respectively, for the first and tenth muscle contractions (Figure 4B). Consequently, C_60_FAS treatment resulted in increases of 15–18 ± 1%, 12–14 ± 1%, and 18–29 ± 2% on days 15, 30, and 45, respectively, compared to the injury group.

Notably, administration of C_60_FAS at a daily oral dose of 1 mg/kg over a 30-day recovery period significantly enhanced the functional activity of the rat *musculus gastrocnemius* (a fast skeletal muscle) following neurogenic atrophy induced by sciatic nerve injury. Specifically, integrated muscle power increased by 13 ± 1% for both the first and tenth contractions compared to the injury group [40], consistent with results observed for the rat *musculus soleus* (a slow skeletal muscle).

Changes in maximum contraction force serve as a marker of general muscular system dysfunction. These changes may be associated with both neuronal disturbances and myopathic factors related to the studied pathology [40].

The maximum force response of the *musculus soleus* on days 15, 30, and 45 after nerve injury reduced to 0.76 ± 0.05 N and 0.51 ± 0.04 N, 0.78 ± 0.06 N and 0.52 ± 0.04 N, and 0.81 ± 0.06 N and 0.61 ± 0.05 N, for the first and tenth muscle contractions, respectively (control: 0.98 ± 0.07 N) (Figure 4C).

Administration of C_60_FAS increased this parameter to 0.78 ± 0.05 N and 0.62 ± 0.04 N, 0.81 ± 0.06 N and 0.63 ± 0.05 N, and 0.88 ± 0.06 N and 0.82 ± 0.06 N on days 15, 30, and 45, respectively, for the first and tenth contractions (Figure 4C). Consequently, C_60_FAS treatment resulted in increases of 3–18 ± 1%, 4–17 ± 1%, and 8–26 ± 2% on days 15, 30, and 45, respectively, compared to the injury group.

Changes in the minimum muscle contraction force serve as a key marker of dysfunction caused by pathological development in successive contraction acts [43].

The minimum force response of the *musculus soleus* on days 15, 30, and 45 after nerve injury significantly reduced to 0.22 ± 0.01 N and 0.10 ± 0.01 N, 0.38 ± 0.02 N and 0.27 ± 0.02 N, and 0.57 ± 0.04 N and 0.35 ± 0.03 N, for the first and tenth contractions, respectively (control: 0.96 ± 0.07 N) (Figure 4D).

Administration of C_60_FAS increased this parameter to 0.52 ± 0.04 N and 0.38 ± 0.02 N, 0.61 ± 0.05 N and 0.51 ± 0.03 N, and 0.86 ± 0.07 N and 0.68 ± 0.05 N on days 15, 30, and 45, respectively, for the first and tenth contractions (Figure 4D). Consequently, C_60_FAS treatment resulted in increases of 58–74 ± 5%, 38–47 ± 3%, and 34–49 ± 3% on days 15, 30, and 45, respectively, compared to the injury group.

Notably, the significant differences in the maximum and minimum contraction forces of the *musculus soleus* may be associated with increased formation of intramuscular collagen structures induced by neurogenic atrophy, which reduces dynamic muscle contraction parameters, particularly the minimum force, which is the most sensitive [38,39].

The results indicate that nerve damage causes significant disturbances in the functional state of the muscle, evidenced by decreases in integrated power, maximum force, and particularly minimum contraction force. These changes reflect the development of neuropathic and myopathic processes associated with neurogenic atrophy and signify a marked decline in the muscle’s ability to perform repeated contractile actions [44]. Biomechanical analysis revealed an improvement in the contractile function of the *musculus soleus* against the background of neurogenic atrophy and following treatment with C_60_FAS. Specifically, the data demonstrate the long-term efficacy of C_60_FAS, showing a maximal therapeutic effect of 23–35 ± 2% compared to the injury group on day 45 of the experiment. This contraction dynamics of the *musculus soleus* throughout the study is consistent with the slow remodeling processes occurring in denervated skeletal muscle [45].

### 2.3. Biochemical Analysis

Changes in the blood chemical composition of experimental animals during prolonged non-relaxation contractions of the atrophic *musculus soleus* reflect biochemical disturbances [46] that arise as a result of fatigue processes developing in the muscle.

C-reactive protein (CRP) is a primary marker of the inflammatory response in the body. The concentration of CRP in the blood indicates the level of inflammation and tissue damage, as well as the presence of postoperative complications [47].

**Figure 5:**
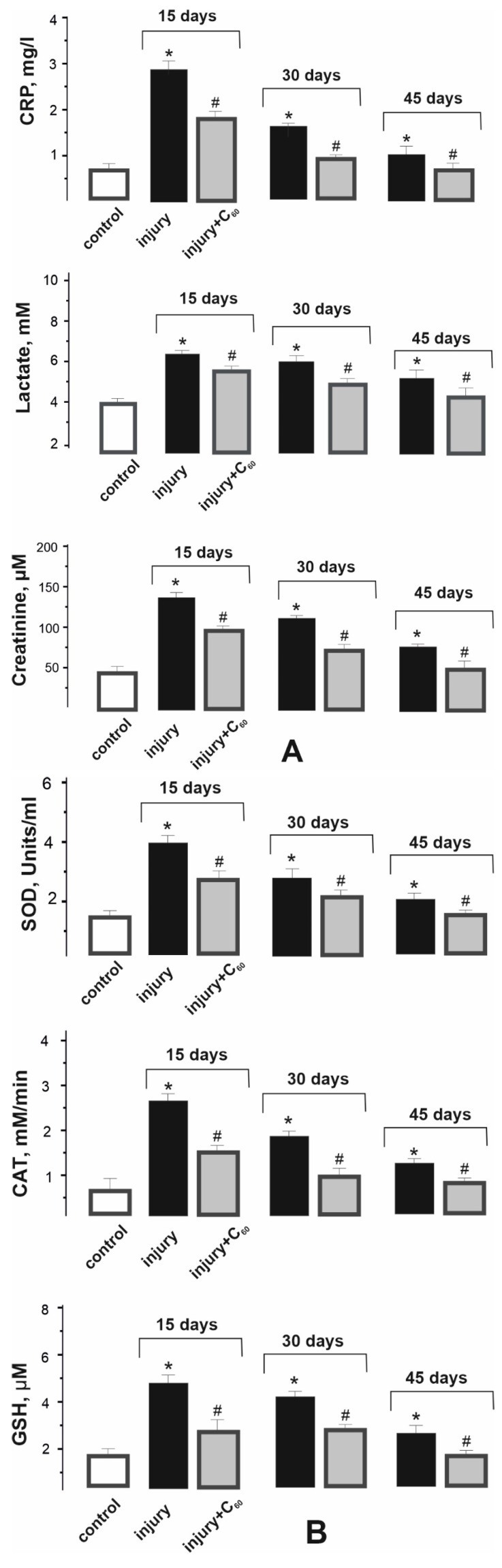
Concentrations of CRP, lactate, and creatinine (**A**), and indicators of pro- and antioxidant balance (SOD, CAT, and GSH) (**B**) in rat blood plasma after the initiation of neurogenic atrophy of the *musculus soleus*: control (white): control group of animals (*p* < 0.05); injury (black) and injury + C_60_ (gray): animals on days 15, 30, and 45 after *nervus ischiadicus* injury and with daily oral administration of C_60_FAS (1 mg/kg), respectively. * *p* < 0.05 relative to the control group; ^#^ *p* < 0.05 relative to the injury group.

An increase in CRP concentration from 0.7 ± 0.1 mg/L in the control group to 2.9 ± 0.2 mg/L on the day 15 after nerve injury indicates ongoing inflammatory processes (Figure 5A). A subsequent reducing to 1.6 ± 0.1 mg/L and 1.0 ± 0.1 mg/L on days 30 and 45, respectively, points to the persistent inflammatory processes in the animals’ bodies.

Administration of C_60_FAS significantly reduced the concentration of CRP in the blood, which was 1.8 ± 0.1, 0.9 ± 0.1, and 0.7 ± 0.1 mg/L on days 15, 30, and 45 of the experiment, respectively (Figure 5A). Thus, the positive effect of C_60_FAS amounted to 30–44 ± 3% throughout the experiment compared with the injury group, confirming the potential of antioxidant C_60_ fullerenes in the therapy of muscle inflammation [37,38,39,40,42]. As can be seen, by the 45th day after the injury, the CRP level in the blood had normalized.

In the active *musculus soleus*, a large amount of lactate accumulates, which, during prolonged non-relaxation contractions, does not have time to oxidize. An increase in lactate concentration in the blood may indicate dysfunction of muscle fibers caused by atrophic changes within them.

The lactate concentration increased from 4.0 ± 0.3 mM in the control group to 6.3 ± 0.5 mM on the 15th day of the experiment. On days 30 and 45, this indicator was 5.9 ± 0.4 mM and 5.0 ± 0.4 mM, respectively (Figure 5A).

Administration of C_60_FAS reduced the blood lactate concentration to 5.2 ± 0.4, 4.9 ± 0.3, and 4.1 ± 0.3 mM on days 15, 30, and 45 of the experiment, respectively (Figure 5A). Thus, the positive effect of C_60_FAS amounted to 17–18 ± 1% compared with the injury group. Notably, by day 45 after nerve injury initiation, the blood lactate concentration had nearly normalized, indicating the ability of water-soluble C_60_ fullerenes to restore its level in the active *musculus soleus***.** For comparison, in the *musculus gastrocnemius* [40], C_60_FAS treatment resulted in a positive effect of 16 ± 1% after 30 days of the experiment.

The creatinine concentration, which forms as a result of damage to intramuscular structures during intensive contractions, increased from 47 ± 2 µM in the control group to 131 ± 5 µM on the 15th day of the experiment. On days 30 and 45, this indicator was 112 ± 5 µM and 74 ± 4 µM, respectively (Figure 5A).

Administration of C_60_AS reduced the blood creatinine concentration to 97 ± 4, 72 ± 3, and 51 ± 2 µM on days 15, 30, and 45 of the experiment, respectively (Figure 5A). Thus, the positive effect of C_60_FAS amounted to 26–36 ± 2% compared with the injury group. Notably, by the 45th day after the initiation of nerve injury, the blood creatinine concentration had nearly normalized.

For comparison, in the case of the *musculus gastrocnemius* [40], the positive effect of C_60_FAS was 34 ± 2% after 30 days of the experiment.

One of the important factors associated with atrophic changes in skeletal muscles after peripheral nerve injury is an imbalance in the redox state. In the muscle, elevated levels of prooxidants and lipid peroxidation are observed, caused by myofibril atrophy. At the same time, no reduction or reversal of this process is observed when using conventional antioxidants [48]. An increase in oxidative stress levels is a pathophysiological feature of neurogenic atrophy, associated in particular with impaired functioning of the ubiquitin–proteasome system, lysosomal autophagy, and mTOR pathways [49].

SOD activity increased from 1.8 ± 0.1 Units/mL in the control to 3.9 ± 0.3, 2.8 ± 0.2, and 2.1 ± 0.2 Units/mL on days 15, 30, and 45 after nerve injury, respectively (Figure 5B).

Administration of C_60_FAS reduced this parameter to 2.6 ± 0.2, 2.1 ± 0.2, and 1.9 ± 0.1 Units/mL on days 15, 30, and 45 of the experiment, respectively (Figure 5B). Thus, the positive effect of C_60_FAS amounted to 14–33 ± 2% compared with the injury group. As can be seen, by day 45 after nerve injury initiation, SOD activity in the blood was almost normalized.

For comparison: in the case of the *musculus gastrocnemius* [40], the positive effect of C_60_FAS was 32 ± 2% after 30 days of the experiment.

Catalase (CAT) activity increased from 0.61 ± 0.05 mM/min in the control group to 2.6 ± 0.2, 1.8 ± 0.1, and 1.3 ± 0.1 mM/min on days 15, 30, and 45 of the experiment, respectively (Figure 5B).

Administration of C_60_FAS reduced this parameter to 1.5 ± 0.1, 1.0 ± 0.1, and 0.80 ± 0.07 mM/min on days 15, 30, and 45, respectively (Figure 5B). Thus, the positive effect of C_60_FAS amounted to 38–44 ± 3% compared with the injury group.

For comparison: in the case of the *musculus gastrocnemius* [40], the positive effect of C_60_FAS was 39 ± 2% after 30 days of the experiment.

The concentration of reduced glutathione (GSH) increased from 1.8 ± 0.1 µM in the control group to 4.8 ± 0.3, 4.1 ± 0.3, and 2.7 ± 0.2 µM on days 15, 30, and 45 of the experiment, respectively (Figure 5B).

Administration of C_60_FAS reduced this parameter to 2.9 ± 0.2, 2.8 ± 0.2, and 1.9 ± 0.1 µM on days 15, 30, and 45 of the experiment, respectively (Figure 5B). Thus, the positive effect of C_60_FAS was 30–40 ± 3% compared to the injury group. As can be seen, by day 45 after nerve injury initiation, SOD activity in the blood had almost normalized.

In summary, the studied biochemical markers of physiological disturbances in muscle tissue [44] exhibit a pronounced increase after the initiation of neurogenic atrophy of the *musculus soleus*. This indicates that the muscular system performs work of an intensity exceeding its physiological capacity, leading to the development of muscle fatigue. At the same time, prolonged administration of C_60_FAS effectively reduces oxidative processes in the damaged muscles by maintaining a balance between pro-oxidants and the antioxidant defense system, which prevents the negative effects of free radicals on cellular and subcellular structures during atrophic processes in rats caused by nerve injury. As can be seen, by the 45th day of the experiment, all the above-mentioned biochemical blood parameters had nearly returned to control values.

It is important to emphasize that the antioxidant effect of C_60_ fullerenes was evaluated by analyzing indicators of the pro- and antioxidant balance (SOD, CAT, and GSH) in rat blood. These markers are widely used in physiological and biomedical studies to assess oxidative stress [46]. The normalization of these biochemical parameters, along with the improvement in muscle functional characteristics, suggests a reduction in oxidative stress following the administration of C_60_FAS.

The positive changes in blood biochemical parameters against the background of neurogenic atrophy of the *musculus soleus* and the action of C_60_FAS are consistent with the concept that denervation atrophy activates systemic metabolic and proteolytic processes [50] and, therefore, can be considered indicative of the systemic response of the organism to neurogenic atrophy and therapeutic intervention.

Data [24,25,26] demonstrate that antioxidants such as vitamin E, B-complex vitamins, pregabalin combined with vitamins and carnosine can reduce inflammation, the severity of degenerative changes in nerve tissue, and oxidative stress. However, the therapeutic effects of these compounds are usually partial and do not ensure complete restoration of muscle function. In contrast to these drugs, the use of water-soluble C_60_ fullerenes was associated with a marked improvement in the contractile function of the *musculus soleus*, near-complete normalization of blood biochemical parameters by the 45th day of the experiment, and a decrease in the degree of fibrosis and damage to muscle fibers.

### 2.4. Histological Analysis

**Figure 6:**
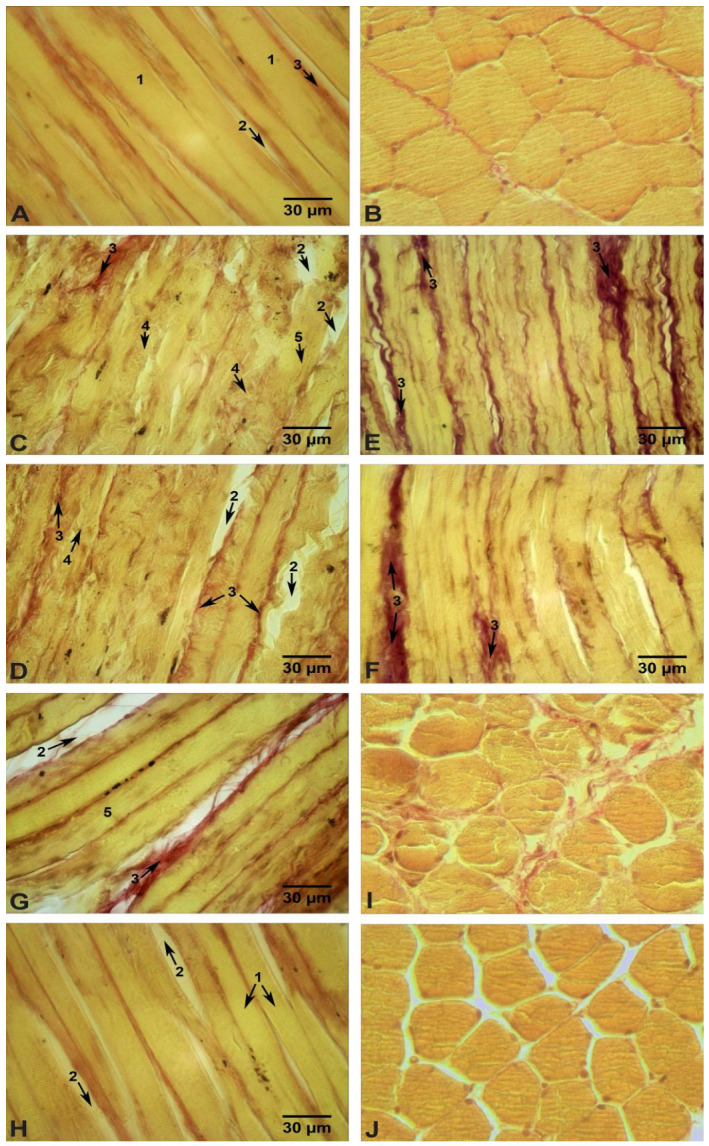
Representative histological images of *musculus soleus*: (**A**,**B**) control; (**C**) 15 days after injury; (**D**) 15 days after injury with C_60_FAS treatment; (**E**) 30 days after injury; (**F**) 30 days after injury with C_60_FAS treatment; (**G**,**I**) 45 days after injury; (**H**,**J**) 45 days after injury with C_60_FAS treatment. (**A**,**C**–**H**)—longitudinal sections; (**B**,**I**,**J**)—transverse sections. (1) transverse striation of the muscle fibers; (2) ground substance of the connective tissue; (3) collagen fibers of the connective tissue; (4) damaged muscle fibers; (5) absence of transverse striation in muscle fibers. Hematoxylin and van Gieson picrofuchsin staining. Scale bar: 30 μm.

In rats of the control group, no deviations from the normal histological structure were observed in the *musculus soleus*. The muscle fibers exhibited transverse striations (Figure 6A, 1). They were surrounded by a small amount of loose connective tissue with a small number of collagen fibers (Figure 6A, 2 and 3; Figure 6B).

15 days after injury, many muscle fibers in the *musculus soleus* are partially damaged and destroyed (Figure 6C, 4). In some muscle fibers, the transverse striations are no longer visible (Figure 6C, 5). The amount of ground substance and collagen fibers in the connective tissue has increased (Figure 6C, 2 and 3).

In rats with injuries that received C_60_FAS, 15 days post-injury, the muscle fibers are partially damaged and degraded (Figure 6D, 4). The connective tissue, including ground substance and collagen fibers, is increased (Figure 6D, 2 and 3).

30 days after injury, the destructive changes in the muscle fibers of the *musculus soleus* decrease. A high level of fibrosis is observed due to the proliferation of collagen fibers (Figure 6E, 3).

In rats with injuries that received C_60_FAS, 30 days after the injury, the destructive changes in the muscle fibers of the *musculus soleus* also decrease. Collagen fibers also increase in number (Figure 6F, 3). However, the degree of fibrosis is lower than in animals that did not receive C_60_FAS.

45 days after injury, the muscle fibers are generally regenerated. However, transverse striation is not visible everywhere (Figure 6G, 5). Between the fibers, the amount of connective tissue and the number of collagen fibers are increased indicating a moderate level of fibrosis (Figure 6G, 2 and 3; Figure 6I).

In animals treated with C_60_FAS, 45 days after injury, the histological structure of the *musculus soleus* is almost the same as in the control (Figure 6H,J). Fibrosis is absent. The amount of ground substance of the connective tissue is slightly increased (Figure 6H, 2).

The data in Table 1 show a decrease in the diameter of muscle fibers and an increase in the proportion of connective tissue compared with the control group. No significant differences were observed on days 15 and 30 between the groups that received and did not receive C_60_FAS. However, on day 45 the proportion of connective tissue in the group treated with C_60_FAS was significantly lower (by 20 ± 2%) than in the untreated group. This, along with a decrease in the degree of pathohistological changes (Figure 6), demonstrates the protective effect of C_60_ fullerenes.

Histological analysis confirms the presence of structural changes in muscle tissue, consistent with the classical model of denervation atrophy. This model involves a reduction in muscle fiber diameter, sarcomere disorganization, and the subsequent replacement of muscle tissue by connective and adipose elements during the prolonged progression of the process [45]. The use of C_60_FAS mitigates destructive changes in muscle fibers and the level of fibrosis in the *musculus soleus*, promotes its regeneration, and exhibits a time-dependent effect. The most significant effects on the morphometric characteristics of the *musculus soleus* were observed on the 45th day after injury (Table 1; Figure 6H,J). These morphological features correspond with changes in biomechanical parameters within the experimental model, demonstrating consistency between structural and functional data.

Notably, prolonged administration of C_60_FAS improved muscle contractile activity and biochemical blood parameters, and also reducing signs of muscle fiber destruction, effects that were particularly evident on day 45 after injury. We hypothesize that this time-dependent effect is associated with the gradual progression of remodeling processes in denervated muscle tissue. Since oxidative stress, inflammation, and fibrosis develop gradually and persist long time after nerve injury, long-term use of C_60_FAS may consistently mitigate these processes, resulting in the accumulation of positive functional and morphological changes during the later stages of recovery.

## 3. Materials and Methods

### 3.1. Material Preparation and Characterization

The method for preparing highly stable and reproducible C_60_FAS was described in detail in [51]. Briefly, it involves transferring C_60_ molecules (Sigma-Aldrich, St. Louis, MO, USA, purity > 99.95%) from toluene into water under ultrasonic treatment (8 Hz, 8 h). The resulting C_60_FAS, with a maximum concentration of 0.15 mg/mL, remains stable for 12–18 months at +4 to 25 °C. It is important to note that the surface hydroxylation of C_60_ molecules (C_60_(OH)_n_) is considered the most likely mechanism responsible for the stabilization of pristine C_60_ fullerenes and their aggregates in water [52].

AFM technique was used to study the structural state of C_60_ fullerenes in aqueous solution. Measurements were performed on dry layers deposited from C_60_FAS onto an atomically smooth mica substrate. AFM measurements were conducted using the “Solver Pro M” system (NT-MDT, Eindhoven, The Netherlands) in the semicontact (tapping) mode with “RTESPA-150” probes (Bruker, Billerica, MA, USA). The size of C_60_ fullerene particles was determined by analyzing profiles along lines passing through the particle peaks.

### 3.2. In Vivo Experiment

Experiments were conducted on male Wistar rats aged 1–2 months (at the end of the study), weighing 110–170 ± 5 g. It should be emphasized that these age limits were selected based on the early onset of fibrosis formation in rats [53]. The rats were housed under controlled environmental conditions (21 °C, 12 h light/12 h dark cycle) with free access to water and a standard diet ad libitum [39]. All procedures with laboratory animals complied with the ARRIVE guidelines. The use of animals was approved by the Biomedical Ethics Committee of the ESC “Institute of Biology and Medicine” at Taras Shevchenko National University of Kyiv (Protocol No. 10, 1 October 2024) and carried out in accordance with Article 26 of the Law of Ukraine “On the Protection of Animals from Cruelty” (No. 3447-IV, 21 February 2006), as well as the European Union Directive of 22 September 2010 (2010/63/EU) on the protection of animals used for scientific purposes.

The sciatic nerve (*nervus ischiadicus*) is a mixed nerve containing sensory, somatic, and autonomic motor axons, primarily originating from the fourth and fifth lumbar segments. The use of a sciatic nerve injury model to study neurogenic atrophy is justified by its surgical accessibility and well-characterized central and peripheral projections [54]. This model involves irreversible disconnection of neurons from their corresponding distal nerve pathways, leading to significant impairment of sensory and motor functions and muscle atrophy [55]. It should also be noted that the development and maintenance of the mitochondrial population in the *nervus ischiadicus* directly depend on the metabolic activity of neuronal cell bodies in the L4–L6 segments. The presence of anterograde transport in the rat *nervus ischiadicus* enables mitochondria to move from the neuronal cell bodies located in the roots toward the synapses. In the event of injury to the *nervus ischiadicus*, the activation of mitochondrial biogenesis in neurons of the L4–L5 roots specifically determines the rate of limb function recovery, making the rat *nervus ischiadicus* an optimal model for studying axonal transport impairment following mechanical injury [56].

The *musculus soleus*, a slow-twitch muscle in rats, undergoes approximately 50% atrophy 14–21 days after hindlimb unloading following sciatic nerve injury [57], making it an optimal model for studying established neurogenic atrophy.

The choice of the *musculus soleus* as a model for studying mechanical injury is based on its unique histochemical profile. Since it consists almost entirely of type I fibers, it allows for the isolated study of reactions characteristic of aerobic metabolism [58,59]. Due to its high mitochondrial content and high capillary density, the *musculus soleus* responds acutely to impaired blood supply, that often accompanies mechanical trauma. This makes it a standard model for investigating oxidative stress. Following injury, alterations in the Krebs cycle and fatty acid metabolism are detected more rapidly in such muscles, which is crucial for understanding the systemic response of the organism to tissue damage and regeneration [60]. Additionally, because of the high myoglobin content in red fibers, its massive release into the bloodstream after injury to the *musculus soleus* serves as an early marker for assessing the progression of the pathological process [61]. While type II fibers degenerate more rapidly following injury, type I fibers persist for a prolonged period, allowing the therapeutic effects of pharmacological agents to be studied.

It is also important to note that one of the primary differences between immobilization models in humans and rodents is the timing of atrophy onset: in rodents, atrophy develops much more rapidly [62].

During the experiments, blood pressure and heart rate were continuously monitored using a PM5000V veterinary patient monitor (Chongqing, China). Euthanasia of animals was conducted by administering an overdose of the anesthetic agent (Zoletil, VIRBAC, Carros, France, 40 mg/kg).

The *nervus ischiadicus* of the experimental animals was transected 10 mm proximal to the bifurcation of the tibial and peroneal nerves [40]. The surgical procedure (axotomy) involved making a skin incision along the femur, extending from the greater trochanter to the popliteal fossa. The muscles (*musculus biceps femoris* and *musculus gluteus superficialis*) were separated along the direction of their fibers to expose the common trunk of the *nervus ischiadicus*. The nerve was carefully freed from the surrounding connective tissue proximal to its bifurcation into the tibial and peroneal nerves [63]. It was then ligated at two sites to prevent spontaneous reconnection, and the segment between the ligatures—a 5–10 mm fragment—was completely excised [64].

The *musculus soleus* was isolated from surrounding tissues, tendons were transected distally, and ventral roots were severed at their spinal cord exit points [37,38].

The animals were randomly assigned to the following experimental groups: control (*n* = 10); injury groups (15 days (*n* = 10), 30 days (*n* = 10), and 45 days (*n* = 10) after sciatic nerve transection); and injury + C_60_ groups (daily oral administration of C_60_FAS at a dose of 1 mg/kg body weight starting immediately after injury induction, for 15 (*n* = 10), 30 (*n* = 10), or 45 (*n* = 10) days). Note that rats in both the injury and control groups received equivalent doses of saline.

The selected C_60_FAS dose was based on previously demonstrated high efficacy in a dose-dependent treatment of various muscle pathologies in vivo [37,38,39,40]. Moreover, the total maximum dose of 45 mg/kg used in this study was substantially lower than the reported LD_50_ value of 721 mg/kg for intraperitoneal administration in mice [65] and was therefore considered safe for biotesting. In addition, C_60_FAS at a concentration of 40 μg/mL was shown to have no genotoxic effect on adult *Drosophila melanogaster* and did not affect reproduction of embryogenesis [66]. Recent studies, conducted by a laboratory accredited by both the FDA and OECD, have further confirmed that C_60_ fullerene is neither toxic nor genotoxic [67,68].

It is also important to note that following oral administration, water-soluble C_60_ fullerenes are absorbed in the gastrointestinal tract and distributed to various organs and tissues [69].

In summary, the selected C_60_FAS dose should not considered universally optimal for all pathological conditions. Rather, the efficacy of the nanostructure may depend on the nature of the disease, treatment duration, route of administration, and the characteristics of the affected tissue.

### 3.3. Biomechanical and Biochemical Analyses

Changes in the contractile force of the *musculus soleus* were measured using tensometric sensors. The external load applied to the muscle was controlled by a mechanostimulation system. The force sensor and mechanostimulator were connected via a feedback loop, forming a servo-controlled system that enabled precise, discrete regulation of muscle force and length at any point during *musculus soleus* contraction [37,38,39]. The isolated nervus ischiadicus was mounted on a bipolar platinum wire electrode for subsequent electrical stimulation, while the muscle tendon was attached to a tensometric apparatus [40]. The edges of the skin surrounding the incision were sutured to the frame of the apparatus, forming a small chamber containing the muscle and nerve, which was then filled with vaseline oil. Electrical stimulation was performed using rectangular pulses of 2 ms duration at a frequency of 50 Hz, generated by an Aurora Scientific ASI 402A pulse generator (Lafayette, IN, USA). The total duration of the modulated stimulation signal was 10 s.

The study of the following mechanokinetic parameters of *musculus soleus* contraction, as markers of the physiological state of the neuromuscular system for animal groups injury and injury + C_60_ [35,36,37,38], was conducted on days 15, 30, and 45 after nerve transection: the time of onset of muscle force response (t_start_), triggered by stimulation pulses; the integrated muscle power (S; this parameter was calculated as the area under the force curve using Origin 9.4 software (Northampton, MA, USA)); the level of maximum muscle contraction force (F_max_); the level of minimum muscle contraction force (F_min_).

Biochemical indicators such as CRP, lactate, and creatinine concentrations, as well as parameters of pro- and antioxidant balance—including the activities of SOD and CAT, and the concentration of GSH—were determined in rat blood on days 15, 30, and 45 following neurogenic atrophy of the *musculus soleus*. Measurements were performed using clinical diagnostic equipment—biochemical analyzers RNL-200 and JN-1101-TR2 (The Netherlands).

It is important to note that blood biochemical parameters offer several advantages over the analysis of muscle tissue. Blood reflects the systemic nature of pathological processes and allows for the assessment of integrated changes in metabolism, inflammatory responses, and the oxidative status of the entire organism, rather than just local alterations within the muscle. Additionally, biochemical blood markers enable rapid dynamic monitoring, which is especially valuable when evaluating the progression of atrophy and the effectiveness of therapeutic interventions. At the same time, analysis of muscle tissue primarily provides local information regarding structural and morphological changes and does not permit long-term monitoring. Moreover, it may be limited by the heterogeneity of the examined material [70].

### 3.4. Histological Analysis

The samples of *musculus soleus* were fixed in 10% formalin [71], embedded in paraffin, sectioned into 5 µm slices, and stained with hematoxylin and picrofuchsin using van Gieson’s method [72]. Digital microphotographs of the stained sections were captured at ×400 magnification using a computer-assisted image analysis system (consisting of an Olympus BX41 microscope and an Olympus C-5050 Zoom digital camera, Tokyo, Japan). The histological profiles of each sample were evaluated through light microscopy. Additionally, the muscle fiber diameters and the area occupied by connective tissue in the muscle bundles were measured using ImageJ software, 1.54t version (NIH, Bethesda, MD, USA).

### 3.5. Statistical Analysis

Statistical analysis of the results was performed using a mixed-design analysis of variance (ANOVA). Two between-group factors were included: (1) injury, and (2) C_60_FAS treatment (two levels: no treatment and C_60_FAS treatment). Time was treated as a within-group factor with three levels (15, 30, and 45 days after injury induction). The Shapiro–Wilk W-test was used to assess normality, and Levene’s-test was applied to evaluate the equality of variances across groups. Multiple pairwise comparisons between groups and conditions were performed using the Bonferroni post hoc test. Differences were considered statistically significant at *p* < 0.05. Each experimental force curve represents the average of 10 repeated measurements. Biochemical and morphometric measurements were performed at least three times and the results are presented as the mean ± SEM. Statistical analyses were conducted using Statistica 8.0 (Dell, Round Rock, TX, USA).

## 4. Conclusions

The results indicate that injury to the *nervus ischiadicus* causes significant impairments in the functional state of the *musculus soleus*. Additionally, there are elevated levels of muscle damage and inflammation markers in rat blood. Substantial, destructive changes in muscle fibers were also observed throughout the experiment. Daily oral administration of C_60_FAS at a dose of 1 mg/kg during the experimental period in rats with neurogenic atrophy had a positive effect on the aforementioned biomechanical parameters of muscle contraction and biochemical blood indices, as confirmed by histological analysis of muscle tissue too. Based on the obtained data, it can be inferred that C_60_ fullerenes have the potential to reduce or correct pathological conditions of the muscular system that arise during neurogenic atrophy, thereby opening promising prospects for their practical application in biomedicine.

## Figures and Tables

**Table 1:** Morphometrical data of *musculus soleus* in rats after injury and C_60_FAS treatment.

Group	Diameter of Muscle Fibers, µm	Area Occupied by Connective Tissue, µm^2^/µm^2^
control	26.8 ± 1.3	0.031 ± 0.003
15 days after injury	22.3 ± 1.3 *	0.069 ± 0.003 *
15 days after injury + C_60_FAS	22.3 ± 0.9 *	0.068 ± 0.004 *
30 days after injury	18.5 ± 0.8 *	0.125 ± 0.011 *
30 days after injury + C_60_FAS	18.4 ± 1.1 *	0.120 ± 0.011 *
45 days after injury	17.7 ± 0.9 *	0.095 ± 0.008 *
45 days after injury + C_60_FAS	18.6 ± 1.0 *^#^	0.076 ± 0.006 *^#^

*p* < 0.05 (control); * *p* < 0.05 compared with the control group; ^#^ *p* < 0.05 compared with the injury group.

## Data Availability

The original contributions presented in this study are included in the article. Further inquiries can be directed to the corresponding author.
